# Long noncoding RNA, *CCDC26*, controls myeloid leukemia cell growth through regulation of *KIT* expression

**DOI:** 10.1186/s12943-015-0364-7

**Published:** 2015-04-19

**Authors:** Tetsuo Hirano, Ryoko Yoshikawa, Hironori Harada, Yuka Harada, Atsuhiko Ishida, Takeshi Yamazaki

**Affiliations:** Domain of Life Sciences, Graduate School of Integrated Arts and Sciences, Hiroshima University, 1-7-1 Kagamiyama, Higashihiroshima, Hiroshima 739-8521 Japan; Department of Hematology, Juntendo University School of Medicine, 2-1-1 Hongo, Bunkyo-ku, Tokyo 113-8421 Japan

**Keywords:** Long noncoding RNA, Myeloid leukemia cells, Cell survival, Receptor tyrosine kinase, shRNA-induced transcriptional gene suppression, Stem cell factor, Serum depletion

## Abstract

**Background:**

Accumulating evidence suggests that some long noncoding RNAs (lncRNAs) are involved in certain diseases, such as cancer. The lncRNA, *CCDC26*, is related to childhood acute myeloid leukemia (AML) because its copy number is altered in AML patients.

**Results:**

We found that *CCDC26* transcripts were abundant in the nuclear fraction of K562 human myeloid leukemia cells. To examine the function of *CCDC26*, gene knockdown (KD) was performed using short hairpin RNAs (shRNAs), and four KD clones, in which *CCDC26* expression was suppressed to 1% of its normal level, were isolated. This down-regulation included suppression of *CCDC26* intron-containing transcripts (the *CCDC26* precursor mRNA), indicating that transcriptional gene suppression (TGS), not post-transcriptional suppression, was occurring. The shRNA targeting one of the two *CCDC26* splice variants also suppressed the other splice variant, which is further evidence for TGS. Growth rates of KD clones were reduced compared with non-KD control cells in media containing normal or high serum concentrations. In contrast, enhanced growth rates in media containing much lower serum concentrations and increased survival periods after serum withdrawal were observed for KD clones. DNA microarray and quantitative polymerase chain reaction screening for differentially expressed genes between KD clones and non-KD control cells revealed significant up-regulation of the tyrosine kinase receptor, *KIT*, hyperactive mutations of which are often found in AML. Treatment of KD clones with ISCK03, a KIT-specific inhibitor, eliminated the increased survival of KD clones in the absence of serum.

**Conclusions:**

We suggest that *CCDC26* controls growth of myeloid leukemia cells through regulation of *KIT* expression. A KIT inhibitor might be an effective treatment against the forms of AML in which *CCDC26* is altered.

**Electronic supplementary material:**

The online version of this article (doi:10.1186/s12943-015-0364-7) contains supplementary material, which is available to authorized users.

## Background

Noncoding RNAs (ncRNAs) are usually transcribed by RNA polymerase II and have intrinsic functions without being translated into polypeptides [1-4]. MicroRNAs (miRNAs), are important ncRNAs of approximately 20 bp that silence specific target genes [5-9], while long noncoding RNAs (lncRNAs) are between 200 bp and several kb and have numerous roles in cellular functions and gene regulation [10]. However, apart from a few examples, the molecular mechanisms by which most lncRNAs function remain to be fully elucidated [11,12]. Accumulating evidence suggests that some lncRNAs are involved in diseases, such as cancer [13,14]; therefore, it is important to understand their roles to facilitate the search for new therapeutic targets and to design new diagnostic methods.

Acute myeloid leukemia (AML) is a disease characterized by mutations in a set of genes [15-17]. These genes can be divided into two classes. One class includes genes related to cell differentiation, *HOXA9*, *AML1*, *MLL* and *RAR*α, and the other consists of genes related to proliferation or survival of cells and includes *FLT3*, *ABL*, *RAS* and *KIT*. Mutations in genes of both classes need to occur to give rise to AML [15,18]. Fusion of *BCR* and *ABL* (*BCR*/*ABL*) is well known to be sufficient to cause chronic myeloid leukemia (CML) [19]. However, acute blastic crisis of CML is often accompanied by mutation of genes that are also mutated in *AML*, such as *HOXA9* or *AML1* [20,21]. Thus, hematopoietic tyrosine kinases, including *FLT3*, *ABL*, *RAS* and *KIT*, seem to have a similar role in AML and CML [22]. Considering the importance of imatinib, an ABL tyrosine kinase inhibitor used in the treatment of *BCR/ABL*-positive CML, inhibitors against other tyrosine kinases are likely to become increasingly important for the treatment of AML [19,23]. KIT is a receptor tyrosine kinase that is considered to have a role in AML because of its frequent up-regulation in patients. Expression of *KIT* in leukemia stem cells (LSCs) from pediatric AML patients who relapsed after chemotherapy was increased compared with that in patients who did not relapse [24].

The function of the lncRNA, *CCDC26*, is not known. There is no evidence for a functional CCDC26 protein; moreover, the hypothetical 109 amino acid protein encoded in its mRNA has no homology with known proteins [25]. Despite the ambiguous nature of *CCDC26*, several lines of evidence support a relationship of *CCDC26* with tumors, including AML. Radtke and colleagues investigated copy number alterations (CNAs) in pediatric AML genomes and found that the most common CNA is a low burden increase of a region within the *CCDC26* locus [26]. Studies by ourselves and others have shown that part of or the entire *CCDC26* gene is often amplified in AML cells harboring aberrant double minute chromosomes [27-29]. Others suggest that sensitivity of AML cells to anticancer drugs, including retinoic acid, is lost with integration of retroviral DNA into the *CCDC26* locus [30]. Linkage of *CCDC26* with tumors, including low-grade glioma, was also suggested by genome wide association studies [31].

In this paper, we demonstrate that knockdown of *CCDC26* in CML-derived K562 cells results in transcriptionally-altered expression of several genes, including activation of *KIT*, and prolonged cell survival under low or no serum conditions. ISCK03, a KIT inhibitor, abolished this prolonged survival. These results provide evidence for a new role of *CCDC26* in myeloid leukemia through the regulation of a set of genes that includes *KIT*.

## Results

### Expression of CCDC26 in various leukemia cell lines

**Figure 1:**
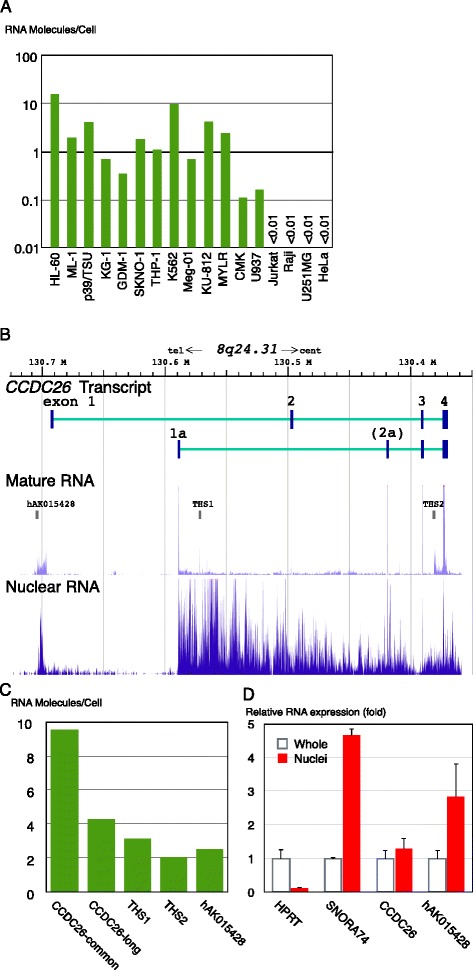
Expression of *CCDC26* ncRNA. **A**: Estimated absolute number of *CCDC26* mRNA molecules per cell in representative hematopoietic and nonhematopoietic cell lines, shown in the log scale. The absolute number of mRNA molecules per K562 cell of *HPRT1*, a house keeping gene, and *BCR/ABL*, an oncogene expressed in K562 cells, was measured as 21+/−5 and 45+/14, respectively. **B**: A map of human chromosome 8q24, showing the location of the *CCDC26* mRNA long and short variants (Genbank accession numbers: NR_130918 and NR_130917, respectively), other minor variants (NR_130919 and NR_130920) containing exon 2A, transcripts including hAK015428, transcriptional hot site 1 (THS1) and THS2, and scores of nuclear and whole cell transcripts in K562 cells. These scores are reprinted from the Human Feb. 2009 (GRCh37/hg19) assembly of the UCSC Genome Browser (http://genome.ucsc.edu/). **C**: Estimated absolute number of *CCDC26*, intron THS1, THS2 and hAK015428 transcripts in a K562 cell. **D**: Quantification of RNA in isolated nuclei and whole cells by quantitative PCR. For each assay, the same amount of RNA was used to synthesize cDNA. The value for *HPRT* in whole cell RNA was used as a standard. *SNORA74*, a snoRNA strictly located in nuclei, was used as a control for purity of the nuclear fraction.

To investigate the spectrum of cells that express *CCDC26*, the absolute number of RNA molecules in a cell was determined by quantitative polymerase chain reaction (PCR) in several representative leukemia and non-leukemia cancer cell lines. The results are summarized in Figure 1A. *CCDC26* was expressed in cells derived from AML (HL-60, ML-1, 039/TSU, KG-1, GDM-1, SKNO-1), acute monocytic leukemia (THP-1) and CML (K562, Meg-01, KU-812, MYLR). Very low expression (estimated at one molecule per cell, taking into account loss of RNA during preparation from cells) was observed in megakaryoblastic cells derived from leukemia accompanied with Down’s syndrome (CMK) and in monocytic leukemia cells derived from histiocytic lymphoma (U937). No expression was observed in T lymphocytic leukemia cells (Jurkat), Burkitt’s lymphoma cells (Raji) or nonhematopoietic cells, including U251MG (astrocytoma) and HeLa (cervical cancer) cells. These data indicate that expression of *CCDC26* is strictly limited to myeloid cell lines of hematopoietic origin. Among them, the strongest expression of *CCDC26* was observed in the AML cell line, HL-60. HL-60 cells, however, have a recombination upstream of *CCDC26* exon 4 and exons 1 to 3 of the gene are amplified on a double minute chromosome, which is an aberrant ring structure consisting of at least six discontinuous regions spanning 440 Mb of 8q24. This region contains several genes, including *CCDC26* and *MYC*, and several abnormal transcripts, one of which involves *CCDC26* exons fused to other unrelated sequences, have been detected [27]. To avoid influence from such a complicated abnormal structure, we choose the K562 cell line, not HL-60, for further detailed analysis because *CCDC26* is also strongly expressed in this cell line and has no rearrangement in the vicinity of the *CCDC26* locus on 8q24.

### Different transcripts are generated from the CCDC26 locus

K562 is a representative hematopoietic cell line, for which extensive biological information is archived in the Encyclopedia of DNA elements database [32]. According to information in the database, there are several transcriptional hot sites in *CCDC26*, including THS1 and THS2, as indicated in Figure 1B, where transcripts accumulate within introns outside of the known exons. In addition, there is a region designated hAK015428 showing a significant level of transcript in the 5′ region upstream of the first exon (Figure 1B). This region is conserved with the mouse ncRNA, AK015428 (Genbank accession number: AK015428). Indeed, we observed significant numbers of THS1, THS2 and hAK015428 transcripts (Figure 1C). hAK015428 was concentrated in the polyA^+^ fraction and its transcription was in the same direction as that of *CCDC26*; therefore, it seems to be an independent lncRNA (Additional file 1: Figure S1A and our unpublished data). We observed accumulation of *CCDC26* and hAK015428 transcripts in isolated nuclei of K562 cells (Figure 1D and Additional file 1: Figure S1A), suggesting a nuclear function.

### Repression of CCDC26 with short-hairpin knockdown vectors

**Figure 2:**
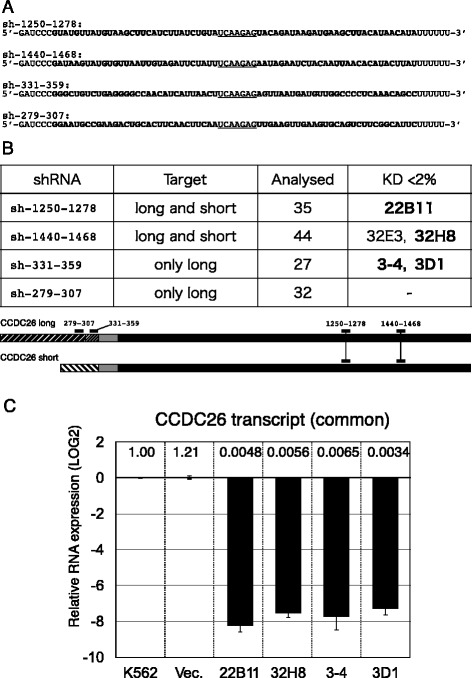
Knockdown of CCDC26 with shRNAs. **A**. Sequences of shRNAs used in this work. The stems formed in hairpin structures are indicated with bold type. The heads of hairpin structures are indicated by underlined sequence. **B**. First screen of *CCDC26* suppression with shRNA vectors. Clones 22B11, 32H8, 3–4 and 3D1 were used in further investigations (bold type). Clone 32E3 was omitted after a second screening. Targets of the shRNAs are mapped on exon structures (1-2-3-4 and 1a-3-4) of the *CCDC26* mRNA variants (see Figure 1B). **C**. Expression of *CCDC26* exon 3–4 in four clones, 3–4, 22B11, 3D1 and 32H8, selected after the second screening. All values were normalized to the expression of *CCDC26* in K562 cells.

**Figure 3:**
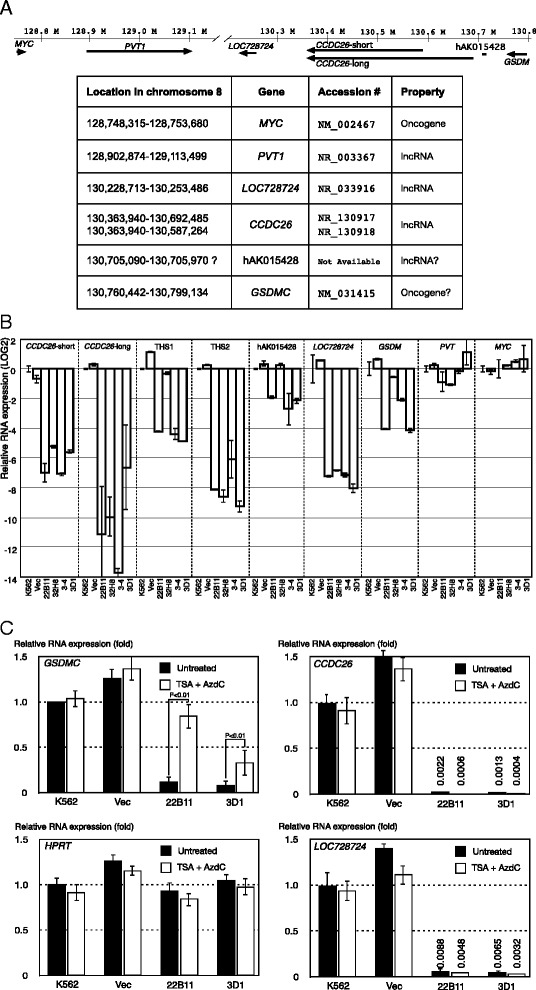
Silencing of the *CCDC26* locus in KD clones. **A:** A map of chromosomal region 8q24 in the vicinity of *CCDC26* (128,800,000–130,800,000; sequence numbering is according to the human genome assembly, GRCh37/hg19). Exon-intron structures are not shown. Arrows indicate the direction of transcription. Location and gene annotation are summarized in the table below. **B**: Expression of *CCDC26*-short, *CCDC26*-long, *LOC728724*, *GSDMC*, THS1, THS2, human AK015428 homolog (hAK015428), *PVTC1* and *MYC*. Graphs are shown in the log2 scale. **C**: Treatment with trichostatin A (TSA) and 5-aza-2′-deoxycytidine (AzdC) prevented the suppression of *GSDMC* but not *CCDC26*. Open and filled boxes indicate with or without TSA + AzdC, respectively.

The function of *CCDC26* was studied by constitutive knockdown using pGER, a short hairpin RNA (shRNA) expression plasmid (Additional file 2: Figure S2A) modified from GeneEraser pGE-1. We designed two shRNAs (sh-1250-1278 and sh-1440-1468) to knock down both the long and short variants of *CCDC26* and two additional shRNAs (sh-331-359 and sh-279-307) to target only the long variant (Figure 2A). Figure 2B shows the results of screening clones for shRNA suppression of *CCDC26* (details of results for all clones are shown in Additional file 2: Figure S2B). Although *CCDC26* suppression failed in most isolated clones, *CCDC26* was suppressed in five clones (22B11, 32H8, 3–4, 3D1 and 32E3) to levels of less than 2% of that in nontransfected K562 cells. After a second screen, we chose four clones (22B11, 32H8, 3–4 and 3D1) for further analysis in which expression of *CCDC26* was repressed to levels of less than 1% of that in control cells (Figure 3C). Clone 32E3 was omitted because the level of *CCDC26* expression was greater than 1% of that in control cells.

### Transcriptional silencing of CCDC26 in knockdown clones

Only one of the two transcript variants (the long variant) was targeted in clones 3–4 and 3D1; however, we observed total knockdown of all *CCDC26* expression. To confirm this observation, we used additional primer sets and we detected that each *CCDC26* mRNA variant was knocked down with similar efficiency. As shown in Figure 1B, there are transcriptionally active sites in *CCDC26* introns. We tested expression from these sites and found that they were also silenced, except for THS2 in knockdown (KD) clone 32H8 (Figure 3B). We also tested the expression of several genes in the vicinity of the *CCDC26* locus (chromosome 8 location; 128,750,000–130,800,000) (Figure 3A). We found efficient silencing of *LOC728724*, a gene independent from *CCDC26*, in all KD clones and partial silencing of *GSDMC* in clones 3–4 and 3D1. However, *PVT1* and *MYC* were not significantly repressed (Figure 3B). Suppression of *GSDMC* in clones 3–4 and 3D1 was partially prevented by treatment with 1.0 μM trichostatin A (TSA), an inhibitor of histone deacetylase, and 1.0 μM 5-aza-2′-deoxycytidine (AzdC), an inhibitor of DNA methyltransferase (DNMT) (Figure 3C), indicating that the silencing of these genes was mediated, at least in part, by epigenetic mechanisms. In contrast, suppression of *CCDC26* was not prevented by treatment with TSA and AzdC (Figure 3C).

### CCDC26 knockdown cells grow slowly in high concentrations of serum and show prolonged survival under serum depletion

**Figure 4:**
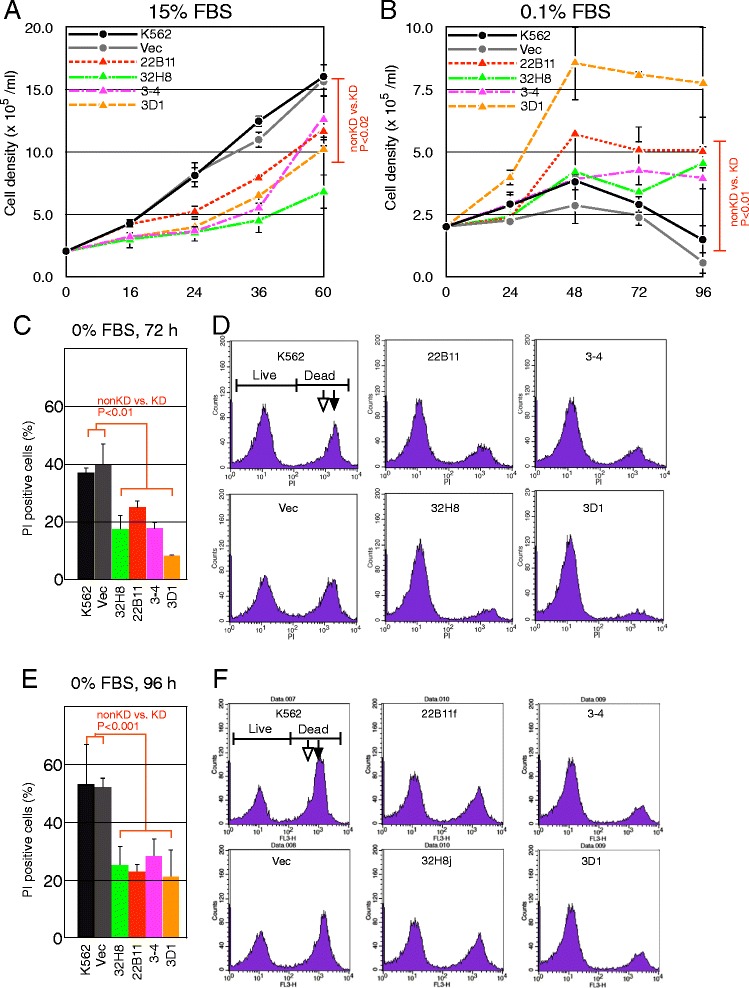
*CCDC26*-KD clones in culture medium containing high or low serum. Growth of *CCDC26*-KD clones in culture medium containing 15% **(A)** or 0.1% FBS **(B)**. The results show the mean of three or four independent experiments. P values for difference between non-KD (K562 and Vec, GER vector transformed) and KD (22B11, 32H8, 3–4 and 3D1) groups are shown. **C**: Number of dead cells after serum depletion measured by PI staining and flow cytometry. The results indicated the average of two independent experiments. P values for differences between non-KD and KD groups are shown. **D**: An example of flow cytometry analysis of cells 72 hours after serum depletion.

Proliferation of K562 cell lines in medium containing 15% fetal bovine serum (FBS) and 0.1% FBS was measured by counting live cells, as determined by trypan blue exclusion. Nontransformed and vector-transformed K562 cells proliferate rapidly with a doubling time of less than 12 hours, whereas KD clones showed prolonged doubling times (approximately 20 hours) (Figure 4A). In media containing 5–10% FBS, similar differences in growth rate between non-KD and KD cells were observed (data not shown). However, more live KD cells were observed compared with non-KD cells after culture for 72 hours under serum starvation conditions (0.1% FBS) (Figure 4B). We detected no significant morphological differences or altered expression of β-globin (a differentiation state marker for K562 cells) between KD clones and non-KD clones indicating no significant difference in the differentiation state of the clones (data not shown).

Less than 10% of non-KD and KD cells died when grown in medium containing high (5% or more) concentrations of FBS up to a density of 1.5 × 10^6^ cells/ml (data not shown). In contrast, a high number of dead cells was observed under low serum conditions after serum starvation for 72 hours or more. Dead cells, as assessed by propidium iodide (PI) uptake, were counted by flow cytometry (FACS) analysis (Figure 4C–F). Significantly fewer dead cells were observed in KD clones compared with non-KD clones at 72 and 96 hours after serum depletion (Figure 4C and E). Figure 4D and F show FACS analysis examples of cells 72 and 96 hours after serum depletion, respectively. The number of dead cells increased with time after serum depletion for all cell lines; however, the cell death rate in KD clones was lower than that in control cells at 96 as well as 72 hours after serum depletion. The right major peak (labeled “DEAD”) corresponding to PI-stained dead cells consists of overlapping double peaks of cells in G1 (open arrow) and G2/M phases (closed arrow). The number of dead cells in the G2/M phase was preferentially decreased in KD cells. This indicates suppression of cell death at a certain point in the cell cycle in KD cells. This result was confirmed by cell-cycle assessment of separated dead and live cells permeabilized and stained with PI. Essentially no difference in distribution of cell cycle-phase was observed between KD and non-KD cells in separated live cells, whereas there was a significant shift in distribution from G1 to S/G2/M in dead cells (Additional file 3: Figure S3A). The rate of cell death for cells grown in 0.1% FBS- or non-FBS-containing medium was also measured by staining with trypan blue. Under each condition, essentially the same result was obtained as above; less cell death was observed in KD clones compared with that in non-KD cells after serum starvation for 72 hours or more (Additional file 3: Figure S3B).

### Identification of genes that are differentially expressed between CCDC26-KD and non-KD cells

**Figure 5:**
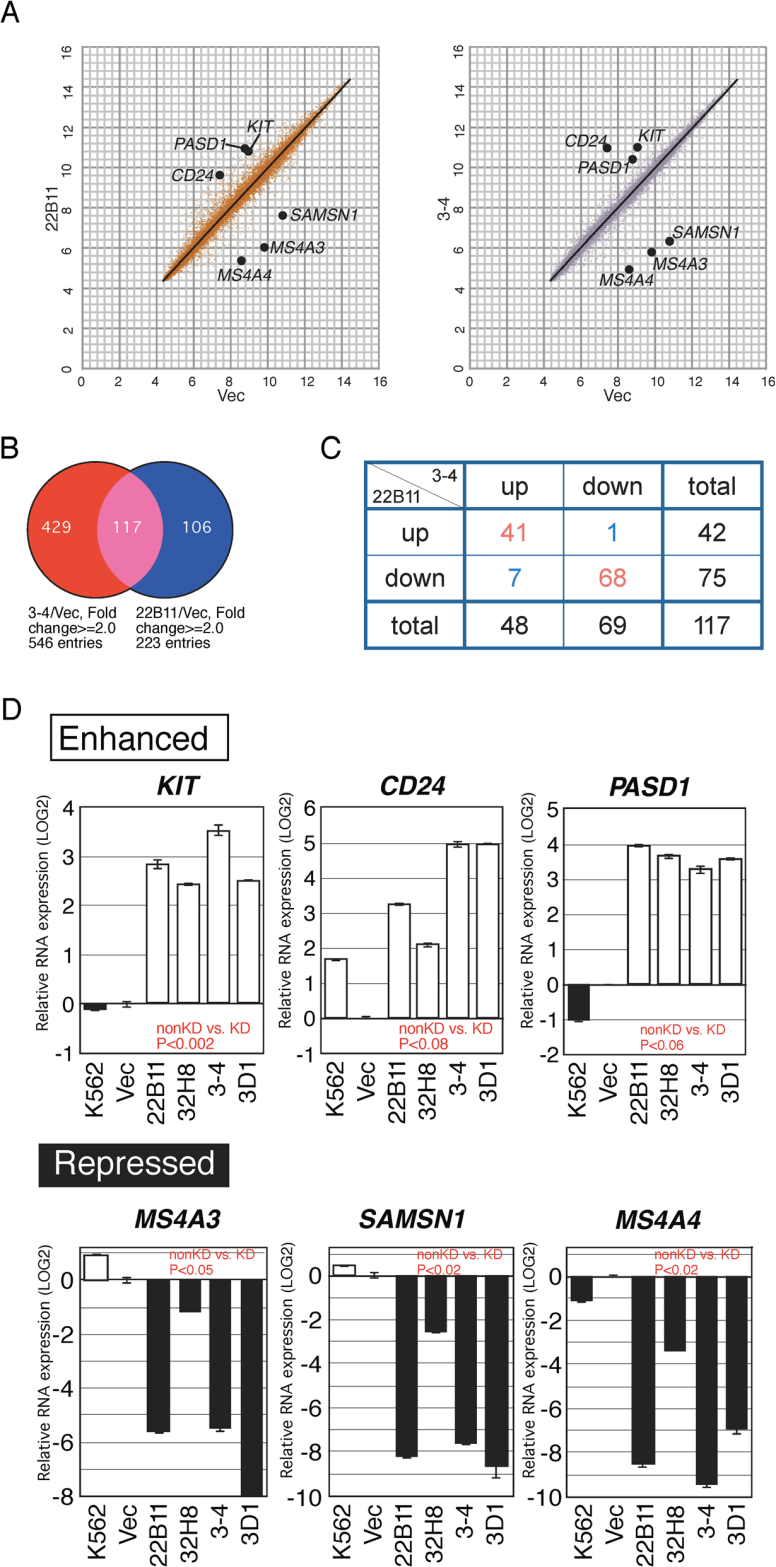
Identification of differentially expressed genes between KD and non-KD cells. **A**: Gene expression levels for KD (22B11 and 3–4) vs. non-KD (Vec: transformed with the empty vector). The six genes in (D) are indicated. **B**: Genes whose expression differed by 2-fold or more between KD and non-KD (Vec.) cells. For 3–4 and 22B11 clones, 429 and 106 genes show altered expression, respectively. Among these genes, 117 showed common differential expression between 3–4 and 22B11 clones. **C**: A cross table of the 117 genes categorized into up or down regulation in each clone. **D**: Expression was enhanced for three genes (*KIT*, *CD24*, *PASD1*) and repressed for three genes (*MS4A3*, *SAMSN1*, *MS4A4*) in *CCDC26*-KD cells. The values were normalized against vector-transformed cells and are shown in the log2 scale. Open and filled boxes indicate enhanced and repressed expression, respectively.

To comprehensively investigate differences of gene expression between KD and non-KD cells, we carried out DNA microarray analysis with RNA purified from nontransfected K562 cells, vector-transformed cells, and KD clones, 3–4 and 22B11 (Figure 5A). Detailed raw results are presented in Additional file 4: Table S1. We extracted 117 genes that showed a 2-fold or greater change in expression level in both KD clones compared with the control cells (Figure 5B and Additional file 5: Table S2). A heat map to visualize their expression is shown in Additional file 6: Figure S4A. Up- or down-regulation of 109 out of 117 genes was detected in both KD cell lines, indicating a consistency of altered gene expression in *CCDC26*-KD cells (P = 1.24E-20, chi-square test for the categorical data in Figure 5C). We chose several most highly differentially expressed candidates for further investigation in the additional KD clones, 3D1 and 32H8, and identified *KIT*, *CD24* and *PASD1* to be significantly activated in all KD clones compared with non-KD clones (Figure 5D upper panel). At the same time, *MS4A3*, *SAMSN1* and *MS4A4* were suppressed in all KD clones (Figure 5D lower panel). Among the six activated and suppressed genes, *KIT* was the strongest candidate to be a target of *CCDC26* because *KIT* is known to be frequently mutated in AML [23]. Moreover, pathway analysis revealed that *KIT*, *CD24* and *MS4A4* form regulatory linkages involving other genes as mediators (Additional file 6: Figure S4B) with *KIT* having a central role in the regulatory network (Additional file 7: Table S3). For confirmation, we quantified other genes frequently altered in AML, including *TET2*, *IDH1*, *IDH2*, *DNMT3A*, *ASXL1*, *EZH-2*, *MLL*, *RUNX*, *CBFB* and *TCF3* [33], but we observed no consistent activation or repression of any of these genes in KD cells (Additional file 8: Figure S5).

### Differential KIT protein levels between CCDC26-KD and non-KD cells

**Figure 6:**
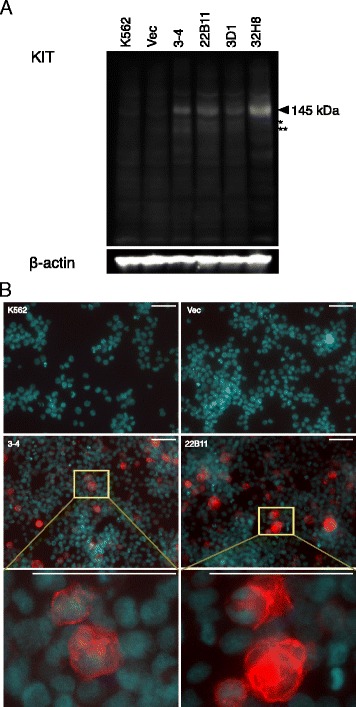
Detecting differential levels of KIT protein. **A**: Western blot analysis for KIT protein in K562, Vec (GER vector transformed), 3–4, 22B11, 3D1 and 32H8 cells. Arrow and asterisks indicate 145 kD membrane bound KIT (arrow), a 120 kD form of KIT (*) and a 100 kD (**) soluble form of KIT. The same blot reprobed with an antibody for beta actin as a loading control is shown below. **B**: Immunocytochemistry for KIT in K562, Vec and KD clones (3–4 and 22B11). Nuclei are counter-stained with Hoechst 33342 (blue). KIT protein is stained red. Higher magnifications for 3–4 and 22B11 are also shown. Scale bars, 50 μm.

The abundance of KIT protein was confirmed by western blot analysis (Figure 6A). The major band of 145 kD, corresponding to the membrane bound form of KIT, was observed in all cell lines. The amount of KIT protein was increased in all KD cells compared with that in non-KD cells, in which a weak KIT signal was observed. Further evidence for enhanced KIT levels was obtained by immunocytochemistry (Figure 6B). In 3–4 and 22B11 KD cells, KIT was concentrated at the plasma membrane and diffusely distributed in the cytoplasm. No signal was detected in nuclei. These observations confirmed the normal localization of KIT in KD cells.

### ISCK03, a tyrosine kinase inhibitor specific to KIT, prevents survival of CCDC26-KD cells under low-serum conditions

**Figure 7:**
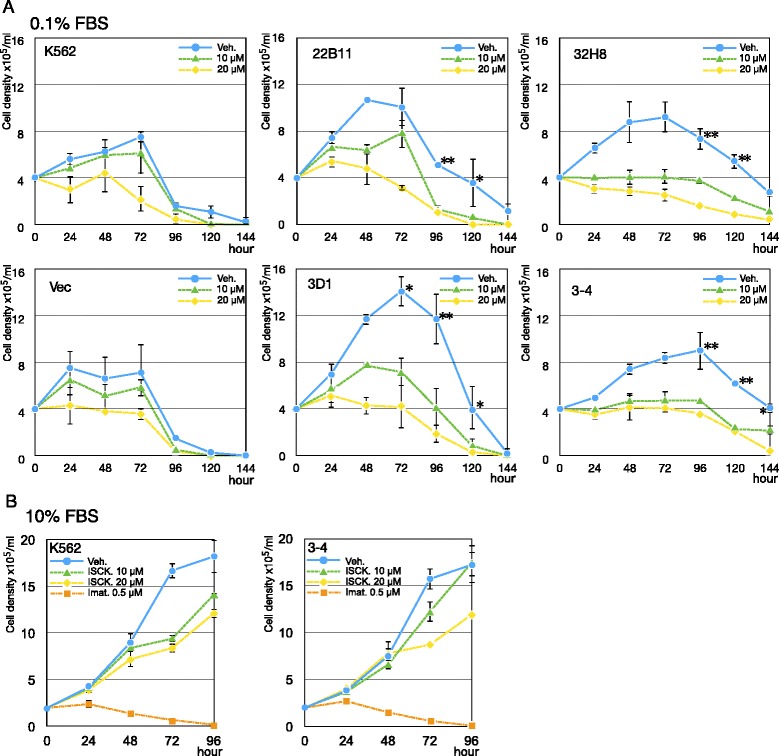
KIT inhibitor prevents survival of *CCDC2*6-KD cells under low serum conditionC **A**: Effect of a tyrosine kinase inhibitor, ISCK03, on the proliferation of K562, Vec (GER vector transformed), and KD clones (22B11, 3D1, 32H8 and 3–4). The numbers of cells in culture medium containing 0.1% FBS with 0 (Veh), 10 μM or 20 μM ISCK03 are shown. *P < 0.05; **P < 0.01 versus corresponding values for Vec. **B**: Number of K562 and KD (3–4) cells in culture medium containing 10% FBS with 0 (veh), 10 μM, 20 μM ISCK03 or 0.5 μM imatinib.

To further investigate the influence of KIT on KD cells, we treated KD cells with ISCK03, a drug that specifically inhibits KIT activity [34]. All treated cells exhibited sensitivity to ISCK03 in a dose-dependent manner (Figure 7A). After ISCK03 treatment, the survival of KD cells was suppressed to the same level as that of non-KD cells. Conversely, ISCK03 treatment had limited effects on the growth of control K562 and KD clone 3–4 cells under high-serum concentration conditions (Figure 7B). Treatment with imatinib, another tyrosine kinase inhibitor specific to ABL but not to KIT, completely abolished the growth of both control K562 and KD cells (Figure 7B). These drugs produced similar growth effects in both control K562 and KD clone 3–4 cells in 10% FBS-containing medium.

## Discussion

We found several regions in the *CCDC26* locus from which transcripts are produced. These transcripts may be independently transcribed or processed from *CCDC26* introns that have been spliced out of its precursor mRNA. The transcripts accumulate in K562 cells but, at present, it is difficult to determine which products are functional among the mature mRNA and intronic *CCDC26* transcripts [35]. However, accumulation of mature *CCDC26* products in the nucleus suggests a functional role [36,37].

An shRNA usually suppresses its target gene by post-transcriptional gene suppression (PTGS), which includes RNA degradation via the RNA-induced silencing complex (RISC). But in some cases, transcriptional gene suppression (TGS) can take place via the RNA-induced transcriptional silencing complex (RITS) [38,39]. In this study, shRNA-mediated PTGS does not account for the suppression of the majority of *CCDC26* transcripts in KD strains (22B11, 32H8, 3–4 and 3D1); partial TGS may have occurred in 32H8 cells because the THS1 transcript was not suppressed in these cells, as shown in Figure 2B. A straightforward explanation in this case is TGS. Moreover, the chromatin range over which TGS occurs is somewhat different among the *CCDC26*-KD clones and 1.0 μM TSA and 1.0 μM AzdC partially prevented the silencing (Figure 3C). Release from silencing by TSA and AzdC was observed at genes located on the borders of the suppressed chromosomal region. This suggests that TGS of these genes by epigenetic mechanisms is accompanied by repressive chromatin modification in the *CCDC26*-KD clones. In contrast, *CCDC26* itself and the adjacent gene, *LOC728724*, remained silent even after treatment with TSA and AzdC (Figure 3C), indicating that TGS of these genes occurs via a different mechanism.

All the KD clones showed lower growth rates than non-KD control K562 cells. It seems likely that *CCDC26* acts as an oncogene to control cell proliferation, either directly or indirectly. Paradoxically, however, the KD clones proliferated more rapidly in low serum conditions compared with non-KD cells. Additionally, the KD clones survived for longer under conditions of very low or no serum compared with non-KD control cells, which died relatively rapidly under these conditions. This observation indicates that suppression of *CCDC26* enables leukemia cells to survive and proliferate despite a severe shortage of growth factors.

DNA microarray and quantitative PCR analysis revealed that the expression of several genes was changed in KD clones. Among them, activation of *KIT* is especially interesting because this gene is over-expressed in many AML patients. Of note, however, activation of KIT protein in KD cells seemed to be stochastic in spite of their monoclonal origin (Figure 6B). It is likely that there are cells that never express *CCDC26* and cells that express more than one molecule, because the number of *CCDC26* transcripts in knockdown cells was calculated at less than one per cell, but the transcript was still detected. This might explain the heterogeneous expression of KIT protein in KD cells, although further investigation is needed to resolve this point. Although the existence of KIT-positive cells *per se* is not a prognostic factor, point mutations in *KIT* that cause enhanced tyrosine kinase activity are frequently accompanied by chromosomal translocation t(8;21) in adult AML, which results in poor prognosis [40]. Other genes, including *CD24* (a sialoglycoprotein expressed on mature granulocytes and B cells), *PASD1* (a transcription factor expressed in diffuse large B-cell lymphoma), *MS4A3* (a hematopoietic cell-specific membrane protein), *SAMSN1* (a protein with an SH3 domain and nuclear localization signals) and *MS4A4* (from the same family as *MS4A3*) are also interesting because of their involvement with tumors but their relationships to myeloid leukemia are not clear. Nevertheless, we cannot exclude the possibility that *CCDC26* regulates cell proliferation or other properties of cells through some of these genes. Overall, we suggest that this ncRNA has novel, previously unknown roles in cellular function. At present, a regulatory function of *CCDC26* has been shown in only a single cell line, K562. Despite our efforts, we have not been successful in suppressing the *CCDC26* transcripts by RNA interference to sufficient levels in cell lines other than K562 for unknown reasons so far (unpublished results). Further study with different approaches including genome editing might be required to confirm its function.

Although *CCDC26* is a low-burden amplified gene expressed in some leukemia cells, amplification occurs partially and does not extend across the whole gene in most cases. This makes it difficult to know whether partial amplification of the gene results in enhancement or loss of its original function. *KIT* is a well-known oncogene; therefore, if over-expression of the incomplete *CCDC26* RNA masks *KIT* function, our findings are consistent with *CCDC26* suppressing some oncogene(s). However, further investigation is required to fully understand this relationship.

LSCs with CD34+ and CD38− surface markers are considered to be a cause of AML recurrence because they have the potential to survive in niche sites that escape the influence of drugs [41]. *CCDC26*-KD cells might share some properties with LSCs, including relatively slow growth and the ability to survive under certain conditions, such as a shortage of growth factors. Constitutive activation of KIT might contribute to survival of these cells by an autocrine mechanism involving stem cell factor, a ligand for KIT [42]. Although LSCs are usually negative for KIT in *de novo* AML [43], the existence of KIT-positive LSCs is related to an increased tendency of pediatric AML recurrence after chemotherapy. This can arise from constitutive activation of KIT protein [44].

## Conclusions

*CCDC26* is thought to transcriptionally regulate a set of genes as well as many lncRNAs [45,46]. The collapse of this regulation might alter the properties of myeloid leukemia, possibly leading to disease progression. Whole or partial amplification of the *CCDC26* locus is associated with pediatric AML and double minute chromosome-positive AML [26,29]. Abnormal *CCDC26* RNA structure could modulate the regulation of *KIT* to induce undesired enhanced expression. Leukemia characterized by mutation of *CCDC26* might be effectively treated by KIT-targeted therapy.

## Methods

### Cell lines

HL-60 cells are described elsewhere [47,48]. K562 and other cells were obtained from ATCC. All cells were maintained in RPMI 1640 medium supplemented with 10% FBS, 100 U/ml penicillin, 100 U/ml streptomycin at 37°C in a humidified incubator with a 5% CO_2_ atmosphere. KD strains derived from K562 were maintained with 0.8–1.0 mg/ml G418 and cultured for at least 12 hours in fresh medium without G418 prior to use in experiments. Cells were counted with a hemocytometer after staining with 0.25% trypan blue. Dead cells were stained blue and live cells excluded the dye. TSA (Sigma-Aldrich, St. Louis, MO, USA) was dissolved in dimethyl sulfoxide (DMSO) at a concentration of 5 mM. The 5 mM TSA solution was diluted 1/5000 in culture medium. AzdC (Sigma-Aldrich) was dissolved in water and added directly to culture medium. Growth of K562 cells was inhibited by 40–60% in media with 1.0 μM TSA and 1.0 μM AzdC. Equivalent concentrations of DMSO were added to control cells without TSA and AzdC.

### Plasmids

The shRNA expression vector, pGER, is a derivative of the pGE-1 GeneEraser plasmid purchased from Stratagene (La Jolla, CA, USA). The original SV40 virus early gene promoter of pGE-1 that drives the G418 resistance marker gene was substituted with the Rous sarcoma virus (RSV) promoter, which is more suitable for leukemia cells. In brief, a 580 bp RSV promoter isolated from pRSV.5(neo) (GenBank: M83237.1) by digestion with *Hind*III and *Nde*I was blunt-end ligated to a 2.6 kb fragment purified from pGE-1 partially digested with *Stu*I and *Pvu*II.

### Isolation of KD clones

Transfection to K562 cells was performed using Dimrie-C (Invitrogen, Carlsbad, CA, USA) according to the manufacturer’s instructions using 0.5 μg of plasmid DNA per 10^5^ cells in 500 μl medium. Cells were plated in methylcellulose based-matrix (ClonaCell; Stem Cell Technologies, Vancouver, Canada) on six-well culture plates to form colonies and were selected with 1.6 mg/ml G418 from 48 hours after transfection. Visible colonies were isolated using a Pipetman under microscopic observation after selection for 6–8 days. Cells were transferred to 96-well plates and were maintained to proliferate in culture medium containing 1.6 mg/ml G418. For the first screening, 10^4^ cells were used for RNA preparation. Clones selected in first screening were subcloned by the limiting dilution method and propagated. RNA was then prepared for a second screening. Colonies of empty pGER transformants were pooled and used as control non-KD cells.

### Quantification of RNA

RNA for quantification was prepared using RNAiso Plus (Takara Bio, Otsu, Japan) according to the manufacturer’s instructions. Synthesis of cDNA was performed using ReverTra Ace qPCR RT Master Mix with gDNA Remover (Toyobo, Osaka, Japan) according to the manufacturer’s instructions. Quantification of cDNAs using primer sets specific to each gene or transcript was performed with the LightCycler system (Roche Diagnostics, Indianapolis, IN, USA). To measure the absolute number of cDNA molecules, known concentrations of PCR product were used to generate a calibration curve. Total RNA in a single cell was estimated at 10 pg (1 mg for 10^8^ cells). In other quantification, we used *HPRT* gene expression as an internal standard. Primer sets used in PCR experiments are listed in Additional file 9: Table S4.

### Isolation of nuclei

Isolation of nuclei was performed according to Spector et al. [49] with slight modification. In brief, 1 × 10^7^ cells were washed once with PBS(−) and suspended in 0.5 ml of buffer A (10 mM Hepes-KOH [pH 8.0], 10 mM KCl, 1.5 mM MgCl_2_, 1 mM dithiothreitol (DTT), 0.5 mM phenylmethylsulfonyl fluoride (PMSF), 0.5 U/μl RNase inhibitor). Cells were incubated on ice for 10 min and then lysed in a Dounce homogenizer. Crude nuclei were collected by centrifugation at 1,300 × g for 5 min and resuspended in 0.5 ml buffer B (0.25 M sucrose, 10 mM Tris–HCl [pH 7.9], 5 mM MgCl_2_, 1 mM DTT, 0.5 mM PMSF). Suspended crude nuclei were centrifuged on a cushion of 0.4 ml buffer C (1.2 M sucrose, 10 mM Tris–HCl [pH 7.9], 5 mM MgCl_2_, 0.1% TritonX-100, 1 mM DTT, 0.5 mM PMSF) at 10,000 × g for 30 min. Precipitated nuclei were resuspended in 0.5 ml buffer B and again centrifuged on a cushion of 0.4 ml buffer C. All procedures were carried out at 0–4°C. The precipitated nuclear fraction was used directly for RNA preparation using RNAiso plus (Takara Bio).

### DNA microarray analysis

Total RNA from each clone was prepared using RNAeasy (Qiagen, Venlo, Netherlands) according to the manufacturer’s instructions. Using an Ambion WT Expression Kit (Ambion, Foster City, CA, USA) and a WT Terminal Labeling Kit (Affymetrix, Santa Clara, CA, USA), 250 ng of RNA was converted to biotin-labeled single stranded cDNA. Fifteen micrograms of single stranded DNA was hybridized to a Human Gene 1.0 ST Array (Affymetrix), followed by staining and washing. Scanning was carried out using a GeneChip Scanner 3000 7G (Affymetrix).

### PI-staining and FACS analysis of dead cells

Cells were washed with PBS and stained in buffer containing 3% FBS, 0.05% NaN_3_ and 2 μg/ml propidium iodide (Sigma-Aldrich) for 15 min at room temperature and analyzed on a FACSCalibur flow cytometer (Beckton Dickinson, Franklin Lakes, NJ, USA).

### Western blot analysis

K562 cells, pGER vector-transformed K562 cells (Vec) and four strains of K562-KD cells were cultured at 37°C in a 5% CO_2_ atmosphere until the logarithmic growth phase. 2 × 10^6^ cells were lysed by sonication in 0.2 ml Laemmli buffer (125 mM Tris–HCl, pH 6.8, 4% sodium dodecyl sulfate (SDS), 100 mM dithiothreitol). Ten microliters of protein extract, equivalent to 1 × 10^5^ cells, were separated on a SDS polyacrylamide gel electrophoresis pre-made 5–20% gradient gel (Wako Pure Chemical Industries, Osaka, Japan) and then transferred to polyvinylidene fluoride membrane (Immobilon-P Transfer Membrane; Millipore, Billerica, MA, USA). The blot was blocked in 7% BlockAce (DS Pharma Biomedical, Osaka Japan) at 4°C for 2 nights to suppress nonspecific signal and then incubated in CanGetSignal solution 1 (Toyobo) with 1/1500 diluted mouse monoclonal anti-KIT antibody (#Ab-81; Cell Signaling Technology, Danvers, MA, USA) and mouse monoclonal anti-beta-actin antibody (#E1C605; Enogene, New York, NY, USA) (to determine loading). After washing, the blot was incubated in CanGetSignal solution 2 (Toyobo) with anti-mouse IgG secondary antibody conjugated with horseradish peroxidase (KPL, Gaithersburg, MD, USA) and then visualized with Chemi-lumi One Super (Nakalai Tesque, Kyoto, Japan). Images were recorded with a Lumino imaging analyzer FAS-1000 (Toyobo).

### Immunocytochemistry

Cells were suspended and fixed in PBS containing 4% paraformaldehyde for 15 min at room temperature. After washing with PBS, cells were suspended in 0.2% Triton/PBS and incubated for 10 min at room temperature. After washing with PBS, cells were spread on glass slides using the Thin-layer cell preparation system Cytospin 4 (Thermo-Fisher Scientific, Waltham, MA, USA). Cells were then incubated with 2% BSA in PBS to block nonspecific signals followed by incubation with 1/100 diluted mouse monoclonal anti-KIT antibody (#Ab-81; Cell Signaling Technology) in CanGetSignal immunostain solution B (Toyobo) overnight at 4°C. Alexa Fluor 546 goat anti-mouse IgG (Invitrogen) was used as a secondary antibody. The specimens were counterstained with 1 μM Hoechst 33428 (AnaSpec, Fremont, CA, USA) and observed under a fluorescence microscope (BA-9000; Keyence, Osaka, Japan).

## Additional files

Additional file 1: Figure S1. Summary of expression from the *CCDC26* locus. Reprinted from the Human Feb. 2009 (GRCh37/hg19) assembly on the UCSC Genome Browser. Scores for K562 cells showing whole cell RNA with polyA, without polyA, cytoplasmic RNA with polyA, without polyA, nuclear RNA with polyA, without polyA, chromatin associated RNA, tri-methylated histone H3K4 and acetylation of histone H3K27. Additional file 2: Figure S2. Summary for *CCDC26* knockdown clones. B: Map of the shRNA vector, pGER. The RSV-promoter (P-RSV) was inserted in place of the original SV40-promoter to drive expression of the neo/kan marker gene in pGE-1. B: An example of detailed expression analysis of KD clones. Values of quantified RNA are shown in the log scale. Averages of clones (AVR) are shown on the right-hand end of each graph. Additional file 3: Figure S3. Cell death of KD cells in low serum conditions. A: Flow cytometry assessment of cell cycle by PI staining of separated live and dead cells at 96 hours after serum depletion. Separation of live and dead cells was performed using Lympholyte Cell Separation Media CL5015 (Cedarlane, Ontario, Canada). Mode values of PI intensity of the live and dead cells are indicated with arrows. B: Cell death rates in medium containing 0.1 or 0% serum. The results at 96 and 122 hours are relatively unclear because cell debris increased counting error. Additional file 4: Table S1. Raw data of the microarray analysis. Additional file 5: Table S2. List of 117 genes with commonly altered expression. Additional file 6: Figure S4. A: Heat map (Blue color for low expression and red color for high expression) and list for the expression levels of the 117 genes. A detailed list of the 117 genes is also shown in Additional file 5: Table S2. B: Pathway analysis for CD24, KIT and MS4A4. Six genes chosen in Figure 5 were analyzed with Pathway analyzing software, KeyMolnet (KMdata, Tokyo, Japan). Known interactions for transmembrane proteins *KIT*, *CD24* and *MS4A4* (red-filled circles) were found. Nuclear and cytoplasmic factors (blue-filled circles) are shown with solid and dashed lines indicating actual binding and regulatory effects, respectively. Additional file 7: Table S3. GO term analysis performed with Ingenuity Pathway analysis software (Qiagen). Additional file 8: Figure S5. Expression of genes frequently altered in AML. The representative expression of genes that are frequently altered in AML patients was measured in KD, Vec (GER vector transformed) and KD clones (22B11, 32H8, 3-4 and 3D1). Additional file 9: Table S4. Primer sets used in this study.

## Abbreviations

AzdC: 5-aza-2′-deoxycytidine
AML: Acute myeloid leukemia
CML: Chronic myeloid leukemia
CNA: Copy number alteration
DNMT: DNA methyltransferase
KD: Knockdown
LSC: Leukemia stem cells
lncRNA: Long noncoding RNA
PTGS: Post-transcriptional gene silencing
PI: Propidium iodide
shRNA: Short hairpin RNA
RSV: Rous sarcoma virus
TGS: Transcriptional gene silencing
TSA: Trichostatin A
