# Supplementary figures and images for: Long noncoding RNA, CCDC26, controls myeloid leukemia cell growth through regulation of KIT expression
Source: Mol Cancer. 2015 Apr 19;14:90. doi: 10.1186/s12943-015-0364-7 (PMC4423487; doi:10.1186/s12943-015-0364-7)

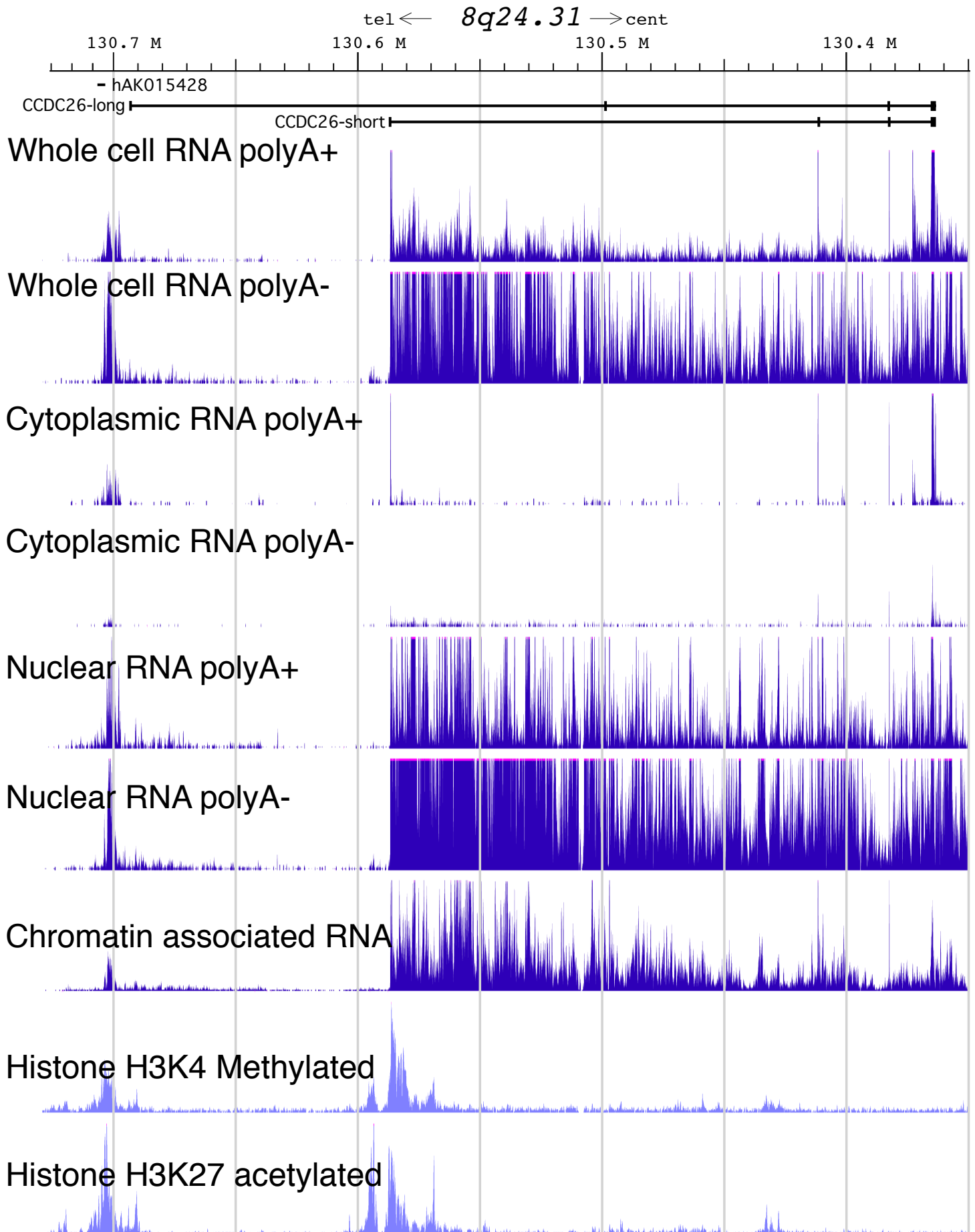

from UCSC Genome Browser on Human Feb. 2009 (GRCh37/hg19) Assembly

Supplement: Additional file 1: Figure S1. — Summary of expression from the CCDC26 locus. Reprinted from the Human Feb. 2009 (GRCh37/hg19) assembly on the UCSC Genome Browser. Scores for K562 cells showing whole cell RNA with polyA, without polyA, cytoplasmic RNA with polyA, without polyA, nuclear RNA with polyA, without polyA, chromatin associated RNA, tri-methylated histone H3K4 and acetylation of histone H3K27. [file 12943_2015_364_MOESM1_ESM.pdf]

Figure S2

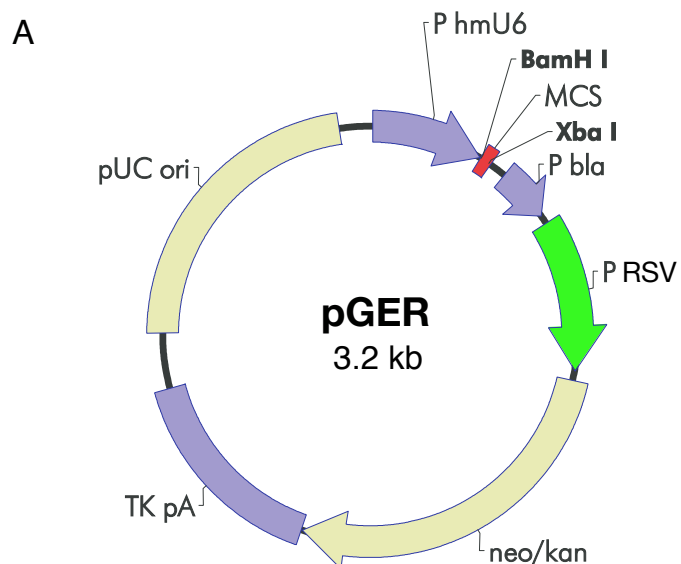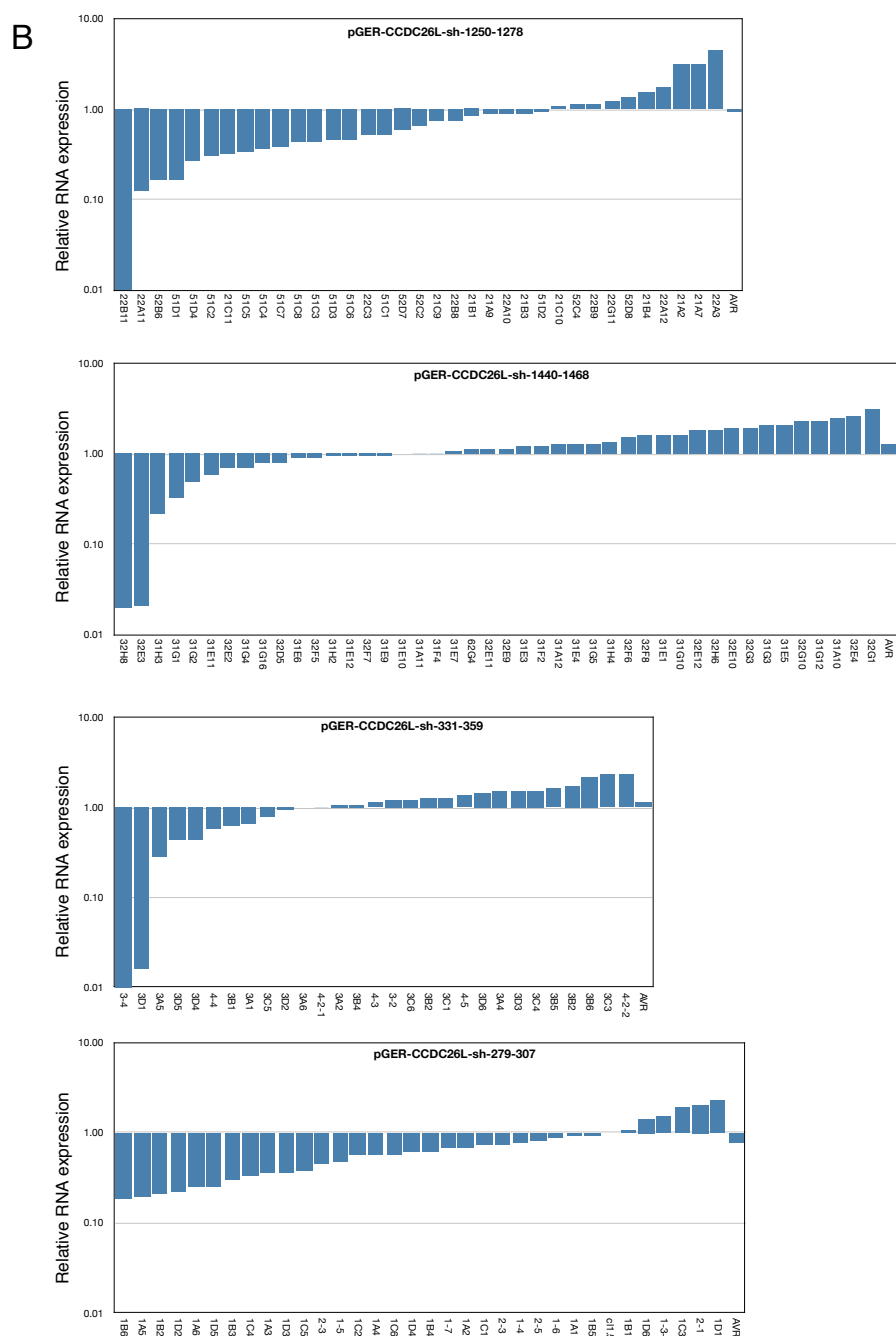

Supplement: Additional file 2: Figure S2. — Summary for CCDC26 knockdown clones. B: Map of the shRNA vector, pGER. The RSV-promoter (P-RSV) was inserted in place of the original SV40-promoter to drive expression of the neo/kan marker gene in pGE-1. B: An example of detailed expression analysis of KD clones. Values of quantified RNA are shown in the log scale. Averages of clones (AVR) are shown on the right-hand end of each graph. [file 12943_2015_364_MOESM2_ESM.pdf]

A

0% FBS, 96 hour

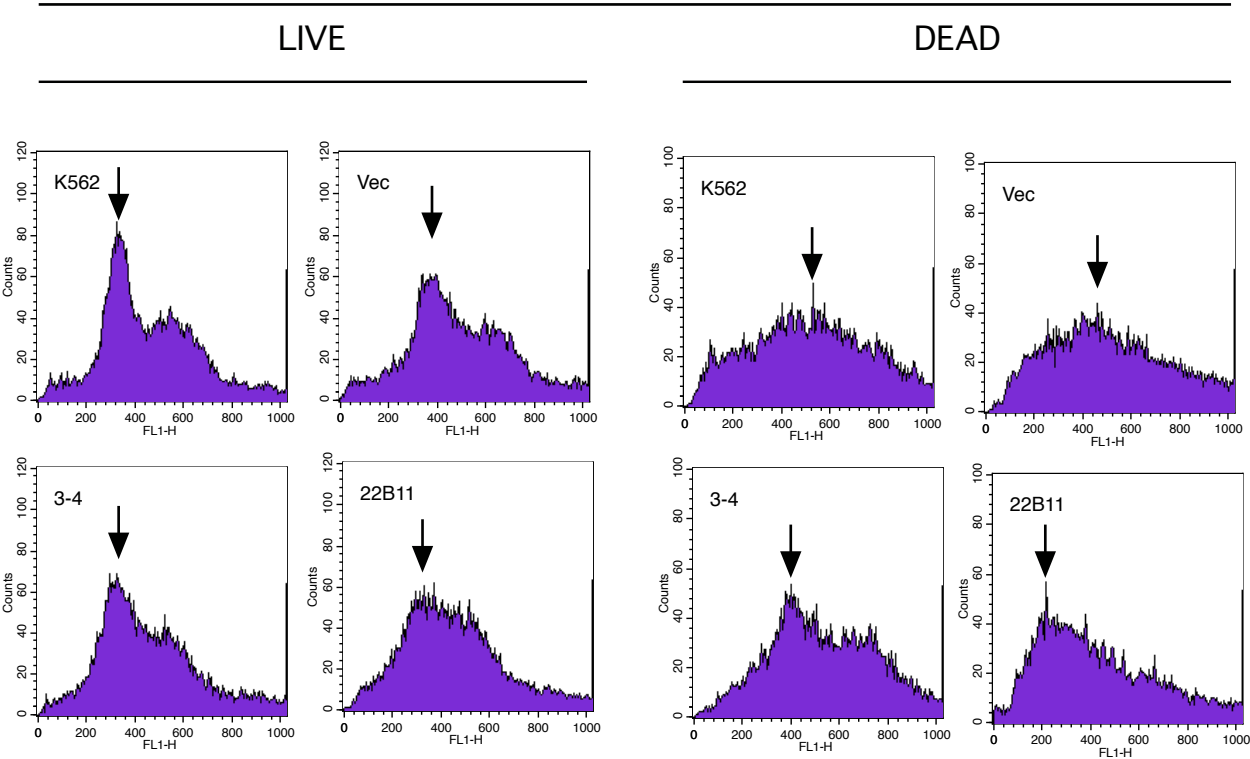

B

0% FBS

0.1% FBS

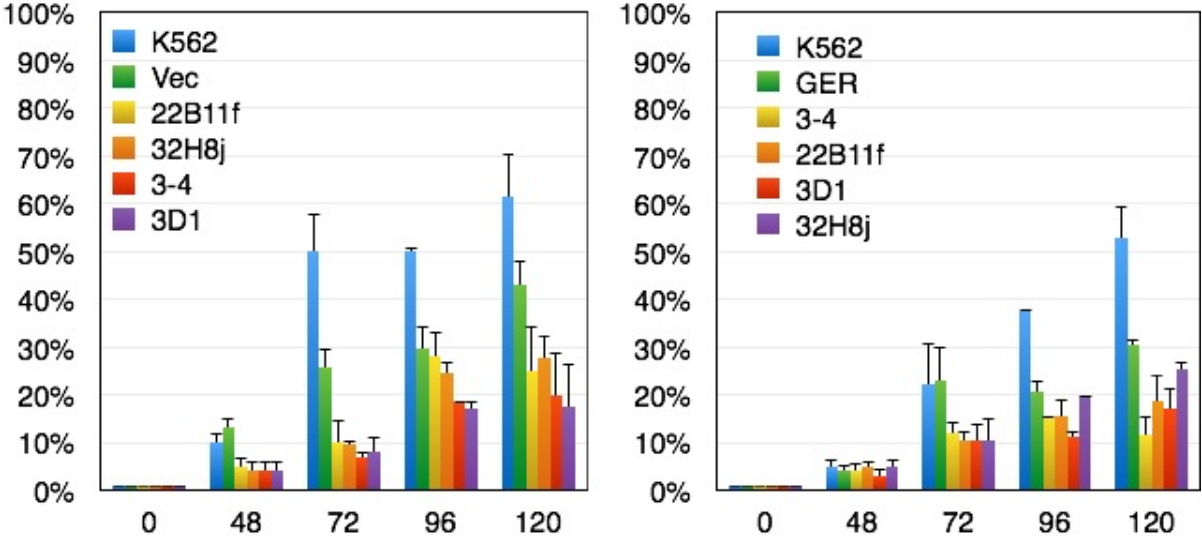

Supplement: Additional file 3: Figure S3. — Cell death of KD cells in low serum conditions. A: Flow cytometry assessment of cell cycle by PI staining of separated live and dead cells at 96 hours after serum depletion. Separation of live and dead cells was performed using Lympholyte Cell Separation Media CL5015 (Cedarlane, Ontario, Canada). Mode values of PI intensity of the live and dead cells are indicated with arrows. B: Cell death rates in medium containing 0.1 or 0% serum. The results at 96 and 122 hours are relatively unclear because cell debris increased counting error. [file 12943_2015_364_MOESM3_ESM.pdf]

Figure S4

A

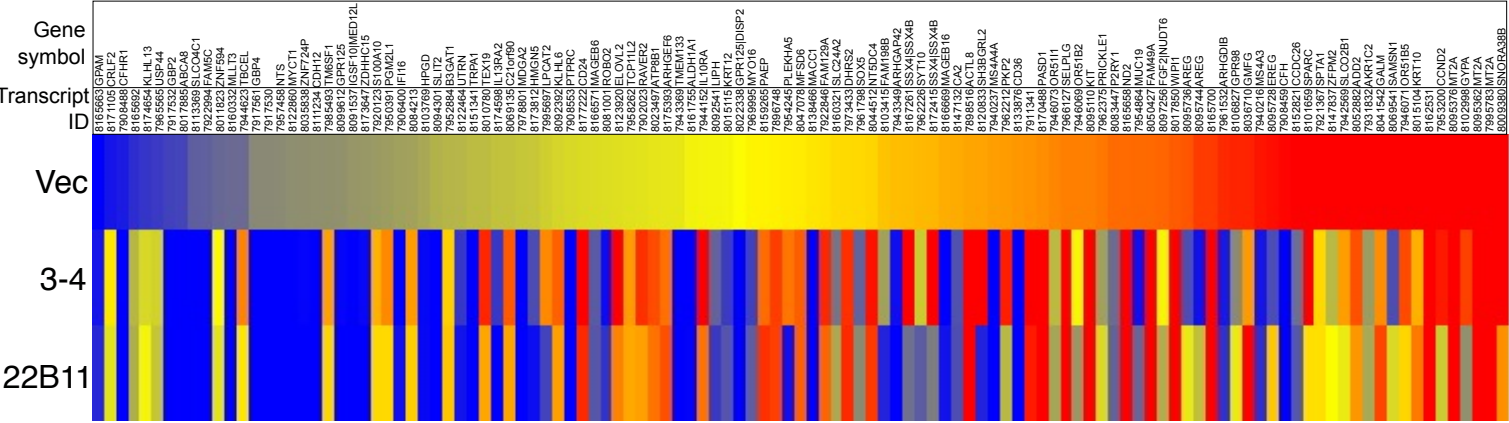

B

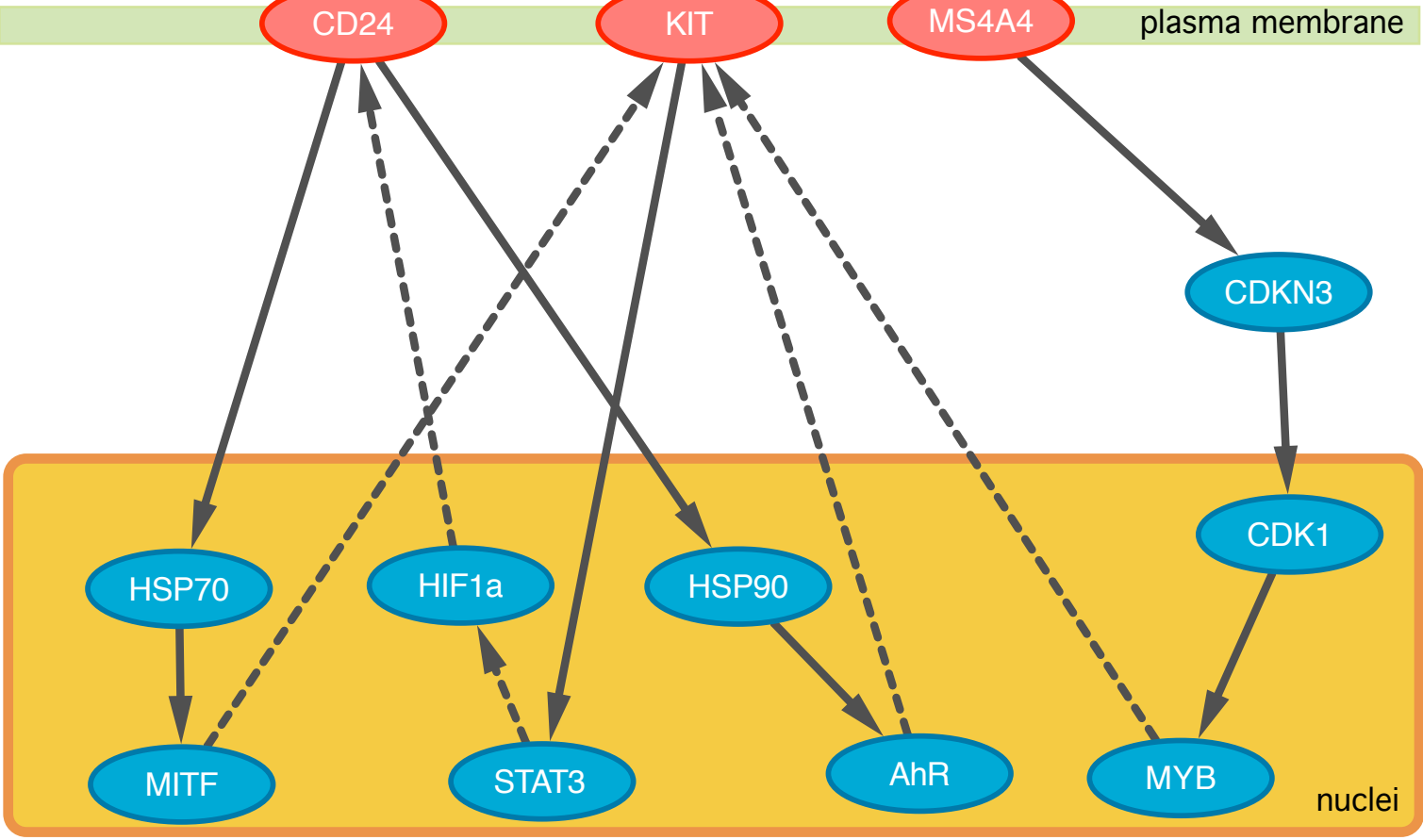

Supplement: Additional file 6: Figure S4. — A: Heat map (Blue color for low expression and red color for high expression) and list for the expression levels of the 117 genes. A detailed list of the 117 genes is also shown in Additional file 5: Table S2. B: Pathway analysis for CD24, KIT and MS4A4. Six genes chosen in Figure 5 were analyzed with Pathway analyzing software, KeyMolnet (KMdata, Tokyo, Japan). Known interactions for transmembrane proteins KIT, CD24 and MS4A4 (red-filled circles) were found. Nuclear and cytoplasmic factors (blue-filled circles) are shown with solid and dashed lines indicating actual binding and regulatory effects, respectively. [file 12943_2015_364_MOESM6_ESM.pdf]

Figure S5

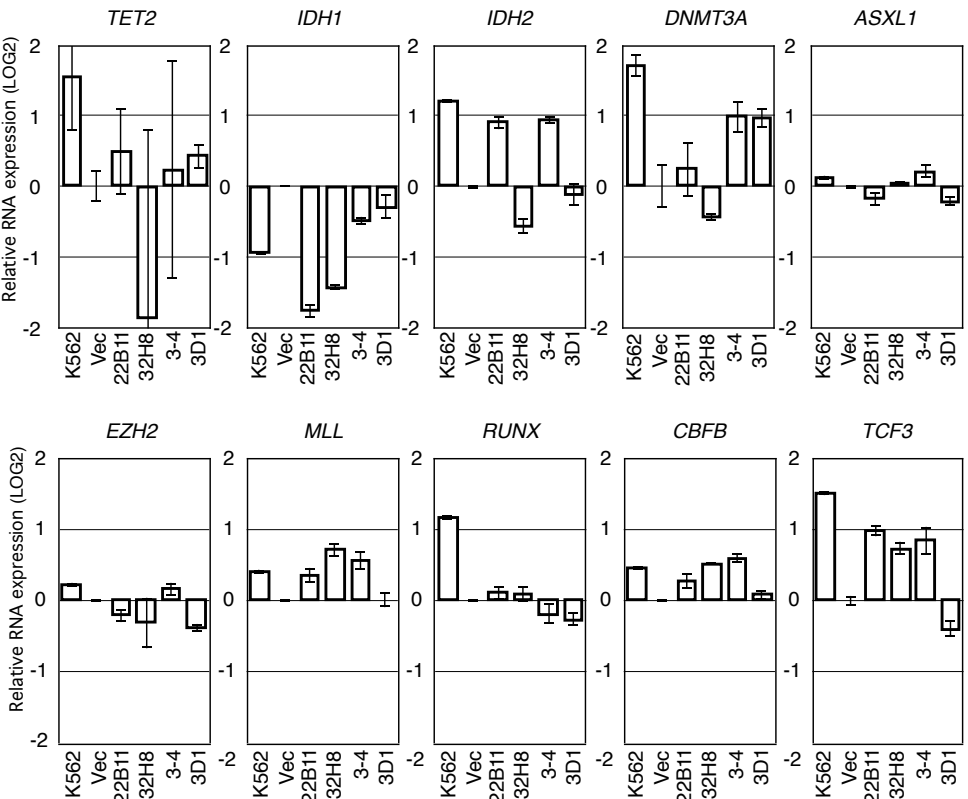

Supplement: Additional file 8: Figure S5. — Expression of genes frequently altered in AML. The representative expression of genes that are frequently altered in AML patients was measured in KD, Vec (GER vector transformed) and KD clones (22B11, 32H8, 3-4 and 3D1). [file 12943_2015_364_MOESM8_ESM.pdf]
