# Supplementary material for: Long noncoding RNA, CCDC26, controls myeloid leukemia cell growth through regulation of KIT expression
Source: Mol Cancer. 2015 Apr 19;14:90. doi: 10.1186/s12943-015-0364-7 (PMC4423487; doi:10.1186/s12943-015-0364-7)
Supplement: Additional file 9: Table S4. — Primer sets used in this study. [file 12943_2015_364_MOESM9_ESM.pdf]

Primer set	Forward	Reverse
CCDC26-common	AGGCCTGAGGAGAGAAGACAC	AGAGCAGCCTGAAAAATGGA
CCDC26-short	ACAGTGCTACTGGCCCATAATCAG	ACAACACATCATAGCCAAATGGAGATT
CCDC26-long	GAGGGGCCAACATCATTAAC	AGAGCAGCCTGAAAAATGGA
THS1	GCGATATGAAGTCAGAGATCCTAGTGG	CACAAGGGGTCAACATGATTGTAAAGT
THS2	GTTAGTCTCAGGTCATGCCAGTCACAG	GACTGGACACCAAGGCTAAGCATCT
hAK015428	AGCACATTTGTGTTTTGGCAAGTT	GCGATACATGCTTCATGTCTTCAG
HPRT	CTTTGCTGACCTGCTGGATT	ACAATCAAGACATTCTTTCCAGTTA
MYC	GCTGCTTAGACGCTGGATTT	CACCGAGTCGTAGTCGAGGT
PVT	TTGGGTCTCCCTATGGAATG	AGACTGTCCACCGCCAAA
LOC728724	CAGCATTTTCCTCCACCCAC	ACTCCATCAGCACGGTGATT
GSDMC	CCAAAATACGAACAAGTTCACATCCTC	GGCCAGGATACTGTAGAACATGACATC
KIT	CACTGTGCATGGCTCTGTTAGAGT	TAGGAGCAGAACGCAGAGAAAATC
CD24	CACTGCTCCTACCCACGC	TGGTGGTGGCATTAGTTGGA
PASD1	TCAACCAAGTGACGCTACAGT	TTCTCTTCATCAGGCAGAAGG
MS4A3	ATTGCACTAGTGGGGACTGC	TCAGCAGTAGAGACACCATGC
SAMSN1	GCCACACACGTCTCAAGTCT	TTACCCAAACCGCCTCCATT
MS4A4	TGGCTGTCATACATTCACATCTG	GCTCATCAGGGCAGTCAGAA
TET2	ACACCTGTCAAGACTCAATATGA	CTGCCACATTAGGACCTGCT
IDH1	AGACGTCCACCAATCCCATT	TGAAGCCAGCCTCAATTGTC
IDH2	TGACCCGTATTATCTGGCAGT	CCAGTGCAGAGTCAATGGTG
DNMT3A	GGTTGTGAGAAGGAATGGGC	CCCTGGTTTTCTTCCACAGC
ASXL1	GCGCGCCTGGTATTAGAAAA	TGATTCGGCCAGGCAGTTTA
EZH2	TCAAGAGGTTCAGACGAGCT	CAAGTCACTGGTCACCGAAC
MLL1	ATCGTCCTCAGCCTCTTCAG	GGGACTTCGCACTCTGACTT
RUNX	CCAACTTCCTCTGCTCCGT	GTTCTTCATGGCTGCGGTAG
CBFB	GCCGCGAGTGTGAGATTAAG	GTTTGTCGCTGTTCTCCCTG
TCF3	ACAAGGAGCTCAGTGACCTC	CTGTGCGACTCAGTGAAGTG
SNORA74	CAGTTGTCAGCTATCCAGGC	ATTGTTTGCACCCAGACCAG
BCR/ABL	ATGCTGACCAACTCGTGTGT	CCCTGAGGCTCAAAGTCAGA
